# Cavitation erosion-corrosion properties of as-cast TC4 and LPBF TC4 in 0.6 mol/L NaCl solution: A comparison investigation

**DOI:** 10.1016/j.ultsonch.2024.106947

**Published:** 2024-06-08

**Authors:** Zhou Yang, Liang Li, Yanxin Qiao, Chengtao Li, Lianmin Zhang, Jie Cui, Dechun Ren, Haibin Ji, Yugui Zheng

**Affiliations:** a School of Materials Science and Engineering, Jiangsu University of Science and Technology, Zhenjiang 212003, China; b CAS Key Laboratory of Nuclear Materials and Safety Assessment, Institute of Metal Research, Chinese Academy of Sciences, Shenyang 110016, China; c Materials Engineering Technology Center, Suzhou Nuclear Power Research Institute, Suzhou 215004, China; d School of Naval Architecture and Ocean Engineering, Jiangsu University of Science and Technology, Zhenjiang 212003, China; e Shi-changxu Innovation Center for Advanced Materials, Institute of Metal Research, Chinese Academy of Sciences, 72 Wenhua Road, Shenyang 110016, China

**Keywords:** Cavitation erosion, Corrosion, Laser powder bed fusion, TC4 alloy, Microstructure, Electrochemical behavior

## Abstract

In this work study, a comparative analysis was undertaken to investigate investigation into the cavitation erosion (CE) and corrosion behavior of laser powder bed fusion (LPBF) TC4 and as-cast TC4 in 0.6 mol/L NaCl solution. Relevant results indicated that LPBF TC4 revealed a rectangular checkerboard-like pattern with a more refined grain size compared to as-cast TC4. Meanwhile, LPBF TC4 surpassed its as-cast counterpart in CE resistance, demonstrating approximately 2.25 times lower cumulative mass loss after 8 h CE. The corrosion potential under alternating CE and quiescence conditions demonstrated that both LPBF TC4 and as-cast TC4 underwent a rapid potential decrease at the initial stages of CE, while a consistent negative shift in corrosion potential was observed with the continuously increasing CE time, indicative of a gradual decline in repassivation ability. The initial surge in corrosion potential during the early CE stages was primarily attributed to accelerated oxygen transfer. As CE progressed, the significant reduction in corrosion potential for both LPBF TC4 and as-cast TC4 was attributed to the breakdown of the passive film. The refined and uniform microstructure in LPBF TC4 effectively suppresses both crack formation and propagation, underscoring the potential of LPBF technology in enhancing the CE resistance of titanium alloys. This work can provide important insights into developing high-quality, reliable, and sustainable CE-resistant materials via LPBF technology.

## Highlights

• Synergistic effect of CE-corrosion on as-cast TC4 and SLM TC4 was quantitatively evaluated.
• Electrochemical mixed potential theory was under discussion in the CE process of as-cast TC4 and SLM TC4.
• SLM TC4 has superior CE resistance compared with as-cast TC4.
• Mechanism of CE damage for as-cast TC4 and SLM TC4 in 3.5 wt% NaCl solution was elucidated.

## 1 Introduction

Cavitation erosion (CE) arises from dynamic pressure fluctuations within a flowing liquid that cause the continuous formation and subsequent collapse of cavitation bubbles [1], [2]. This, in turn, generates shockwaves and micro-jets that impact the surface of materials, leading to surface failures [3], [4]. CE widely occurs on some components working under flowing conditions like propellers, valves, and water pumps, posing significant risks to the safe operation of associated equipment [5], [6], [7]. Titanium alloys, known for their exceptional corrosion resistance and superior mechanical properties, are widely used to fabricate flow components [8]. The surface film of titanium alloys, conferring excellent corrosion resistance, can effectively protect the substrate from corrosion in corrosive solution [9], [10]. Meanwhile, titanium alloys demonstrate superior resistance to CE and find extensive use in the fabrication of various flow components [11], [12]. However, for complex-shaped flow components, titanium alloys, like other materials, often encounter challenges such as low production yields and high manufacturing costs. Consequently, there is a pressing need for the development of new manufacturing and processing techniques.

Laser powder bed fusion (LPBF) stands as a burgeoning additive manufacturing technique distinguished by its ability to produce intricate parts with complex geometries, including intricate internal channels and voids [13], [14]. LPBF technology offers the advantage of producing components in bulk while maintaining both high stability and a lightweight design [15], [16]. Additionally, LPBF can significantly reduce material consumption and production cycle. In recent years, scholars have undertaken comprehensive investigations into the mechanical properties and corrosion performance of LPBF-processed titanium alloys [17], [18]. For instance, Jaroslav *et al.* [19] investigated the corrosion processes of TC4 titanium alloys manufactured by LPBF and electron beam melting (EBM) in a simulated physiological environment. Their findings indicated that both LPBF and EBM samples exhibited typical passivation behavior, surpassing the traditional TC4 alloy in terms of resistance to localized corrosion. Chen *et al.* [20] delved into the anisotropy of LPBF TC4 sheet. Comparing these materials to conventionally rolled plates, there were no significant differences in Young's modulus across all planes. However, electrochemical assessments revealed that LPBF samples outperformed industrial rolled samples in corrosion resistance. Moreover, Zhang *et al.* [21] conducted a comprehensive research on the formation of passive films on the LPBF TC4 alloy surface. This analysis encompassed an examination of both the composition and semiconductor properties of the passive films. Notably, the passive film developed in seawater exhibited typical n-type semiconductor properties and displayed a lower corrosion current density compared to as-cast TC4 alloy, indicating superior corrosion resistance [22]. Collectively, these findings underscore the potential of the LPBF process in fabricating titanium alloys possessing durable mechanical characteristics and exceptional resistance to corrosion.

Not only these improvements, LPBF technology also offers the capacity to optimize material microstructures, thereby augmenting the CE resistance of materials [23]. Song *et al*. [24], for instance, harnessed LPBF technology to process nickel-aluminum bronze (NAB) castings, which resulted in increased material hardness and improved microstructural uniformity. These advancements consequently enhanced the CE resistance of the materials. Similarly, Ding *et al.* [25] conducted a comprehensive analysis of the impact of LPBF process parameters on the erosion rate of 316L stainless steel. Their findings suggested that stainless steel specimens fabricated using LPBF exhibited a reduced number of structural defects, consequently demonstrating superior CE resistance. Furthermore, the study by Zou *et al*. explored the CE behavior of AlSi10Mg alloy when processed using LPBF at various laser scanning speeds [26]. Notably, their research unveiled significant disparities in CE behavior between LPBF-produced samples and traditionally cast samples, LPBF samples demonstrated an exceptionally low cumulative mass loss rate, merely one-tenth of that observed in the cast samples, thus underscoring their superior CE resistance. These results further highlight the potential of utilizing LPBF for manufacturing CE-resistant titanium alloys. Nevertheless, it is imperative to note that there remains a dearth of comprehensive research regarding the CE and corrosion behavior of LPBF titanium alloys. Therefore, embarking on further investigations in this area is crucial, as it promises to elucidate the intricate mechanisms of CE damage in LPBF titanium alloys and contribute significantly to the advancement of novel manufacturing and processing methodologies.

To this end, we employed LPBF technology to fabricate TC4 specimens in this work and aimed to explore the CE-corrosion performance of TC4 alloys manufactured using the LPBF technique. An experimental setup utilizing ultrasonic vibration cavitation was employed to evaluate the CE resistance of LPBF-fabricated TC4. This evaluation involved assessing the mass loss and the evolution of surface CE morphology, which were used to delineate the CE behavior of TC4 specimens produced through LPBF. Conducting ultrasonic research can help us develop high-quality, reliable, and sustainable infrastructure [27], [28], [29], [30], [31], [32], a summary of relevant work is shown in Table 1. Furthermore, electrochemical measurements were conducted to explore the corrosion behavior of LPBF TC4 alloys in a 0.6 mol/L NaCl solution following CE. This study aims to lay the groundwork for the extended application of LPBF and to offer valuable insights in the CE field.

**Table 1:** Examples of some research that advances the SDGs.

**The Focus of the Study**	**Key Points**	**SDGs and Targets**	**Countries of affiliation (authors)**	**Reference**
Enhance the density and mechanical properties of ceramic coating	• Epoxy resin was introduced into the pores and micro-cracks of plasma sprayed ceramic coating	• SDG-9: Ensure sustainable industry, innovation and infrastructure patterns. Target 9.1 • SDG-12: Ensure sustainable consumption and production patterns. Target 12.2	China	[27]
Enhance the resistance of industrial parts to CE	• The Cr-rich diffusion layer was prepared by chromizing process on the surface of the material • The microstructure was more uniform after heat treatment	• SDG-12: Ensure sustainable consumption and production patterns. Target 12.2 • SDG-14: Ensure the sustainable use of the oceans and their resources. Target 14.1	China	[28]
Failure mechanism of polymer coating structure	• Introduction of intermediate coating • Enhancement of elastic deformation ability of polymer	• SDG-12: Ensure sustainable consumption and production patterns. Target 12.2 • SDG-14: Ensure the sustainable use of the oceans and their resources. Target 14.1	China	[29]
The CE resistance of NbN coating in 3.5 wt% NaCl solution	• Ultrasonic cavitation induction device combined with a variety of electrochemical testing methods	• SDG-9: Ensure sustainable industry, innovation and infrastructure patterns. Target 9.1 • SDG-14: Ensure the sustainable use of the oceans and their resources. Target 14.1	China	[30]
Precise spatial control of CE in a vessel phantom	• Effect of cavitation pulse acoustic parameters on CE • Improve the controllability of CE and reduce the erosion depth	• SDG-3: Ensure healthy lives and promote well-being for all at all ages. Target 3.4 • SDG-6: Ensure availability and sustainable management of water and sanitation for all. Target 6.3	China	[31]
Mechanism of action of YAG in the treatment of kidney stones	• Using new experiments and numerical simulations • The cavitation failure mechanism of Ho:YAG LL stone in the process of dust removal is elucidated	• SDG-3: Ensure healthy lives and promote well-being for all at all ages. Target 3.8 • SDG-9: Ensure sustainable industry, innovation and infrastructure patterns. Target 9.1	USA	[32]

## 2 Experimental

### 2.1 Specimen and solution preparation

**Fig. 1:**
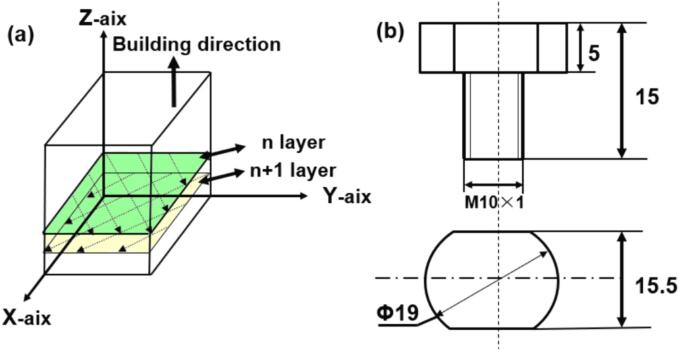
Schematic showing the fabrication process of the LPBF TC4 alloy (a) and dimensions of CE sample (b).

**Fig. 2:**
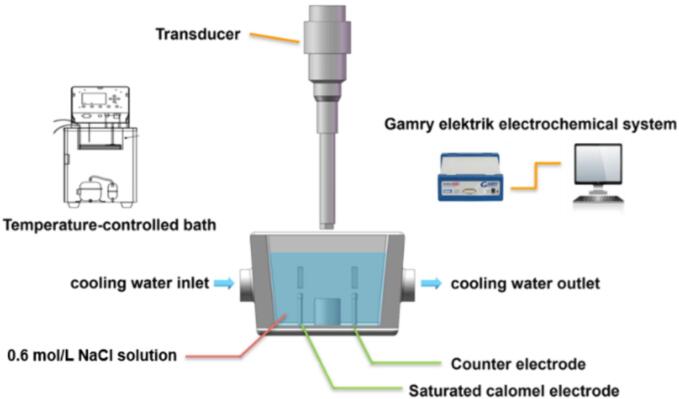
Schematic diagram of CE equipment combining with electrochemical measurement platform.

The LPBF process was carried out on the Concept Laser M2 LPBF equipment. The laser output power was 370 W, the laser scanning speed was 1500 mm/s, the hatch distance was 0.095 mm, the layer thickness was 0.05 mm, and the volume energy density was 208 J·mm^−3^. The samples were made in a protective argon atmosphere and the island exposure strategy was utilized to reduce residual stress during printing. The as-built specimens were annealed at 800 °C for 2 h followed by furnace cooling for stress relieving [33]. The manufacture process of LPBF TC4 alloy was schematically displayed in Fig. 1, and the chemical composition of as-cast and LPBF TC4 alloys was shown in Table 2. The dimensions of the samples utilized in CE and electrochemical measurements were shown in Fig. 1b. The CE tests were carried out utilizing a XOQS-1000 magnetostrictive-induced CE apparatus manufactured by Nanjing Xianou Instrument Manufacturing Co., Ltd., China, as shown in Fig. 2. The apparatus resonated at 20 kHz with a peak-to-peak amplitude of 60 µm based on ASTM standard G32-10. For a detailed description of the experimental methods, readers can refer to previous literature [34]. During the CE tests, the samples were positioned 15 mm deep in a 0.6 mol/L NaCl solution, at a distance of 0.5 mm from the cavitation head. The solution temperature was controlled at 20 ± 2 °C through the circulation of water. The specimens, following various CE durations, underwent deionized cleaning, alcohol degreasing, and subsequent drying using cold air. The polished and corroded samples were weighed using a precision analytical balance (YP10001B, Lichen Instrument Technology Co., Ltd.) with a accuracy of 0.1 mg, and the cumulative mass loss rate (*M*_LR_) was calculated using *E*q*.* (1) [35]:

(1) $${\text{M}}_{\text{LR}}=\frac{{\Delta \text{M}}_{\text{1}}{-\Delta \text{M}}_{\text{2}}{-\Delta \text{M}}_{\text{3}}}{\Delta \text{t}}$$

where Δ*M*_1_, Δ*M*_2_ and Δ*M*_3_ represented the mass change (mg) of the three repeated specimens, Δ*t* was the test duration (h).

**Table 2:** Chemical composition (wt.%) of the as-cast TC4 and LPBF TC4 alloys.

**Alloy**	**Al**	**V**	**Fe**	**C**	**O**	**H**	**N**	**Ti**
As-cast TC4	6.05	4.01	0.36	0.07	0.2	0.011	0.04	Bal.
LPBF TC4	5.63	3.46	0.18	0.03	0.14	0.001	0.01	Bal.

### 2.2 Electrochemical measurements

The electrochemical tests utilized a three-electrode configuration, where the CE specimen functioned as the working electrode, a platinum sheet acted as the auxiliary electrode, and a saturated calomel electrode (SCE) served as the reference electrode. Before conducting the electrochemical measurements, the electrodes were encapsulated with epoxy resin, followed by surface grinding and polishing. For the corrosion potential (*E*_corr_) measurements, a preliminary 800 s static testing was conducted to ensure the relative stability of the system, followed by several alternating cycles of 300 s CE and 300 s quiescence interaction. Prior to potentiodynamic polarization testing, a 1200 s OCP experiment was carried out to ensure the stability of electrochemical testing system. Potentiodynamic polarization (PDP) testing was executed from 300 mV below the *E*_corr_ to 1500 mV vs. SCE at a scanning rate of 0.5 mV/s. Meanwhile, potentiostatic polarization tests at 200 mV vs. SCE were selected from the PDP curve, accompanied by a duration of 1800 s. The Mott-Schottky (M-S) measurements were conducted at a scan rate of 20 mV/s and a test frequency of 1000 Hz, in which the applied potential on the electrode surface ranged from −0.3 V vs. *E*_corr_ to 1 V vs. SCE.

### 2.3 Microstructure and composition characterization

Scanning electron microscopy (SEM, QuattroS, thermovg scientific) was used to observe the morphologies of the corroded samples at different CE times. The grain size and orientation were analysed by electron backscatter diffraction (EBSD) using an Oxford Instruments HKL-EBSD system. The acceleration voltage was 25 kV and the step size was 100 nm. The samples used for this analysis were grounded and electrolytic polished. The electrolytic solution was a mixture of CH_3_COOH (95 vol%) and HClO_4_ (5 vol%) at a temperature of 25 °C, and the applied voltage was 60 V and the current was 0.5 A. After CE for various periods, the surface films developed on the as-cast TC4 and LPBF TC4 alloy were characterized using X-ray photoelectron spectroscopy (XPS, ESCALAB 250Xi, ThermoFisher Scientific, Waltham, MA, USA) at an Al K_α_ radiation of 1486.6 eV. Thermo Avantage software was used to analyze the XPS results. The nanoindentation curves of the LPBF TC4 and as-cast TC4 alloys were conducted using a nano indenter (CSM NHT2, Anton Paar) on the surface. The peak load applied was 20 mN at a loading/unloading rate of 40 mN/min.

## 3 Results and discussion

### 3.1 Microstructure characterization

**Fig. 3:**
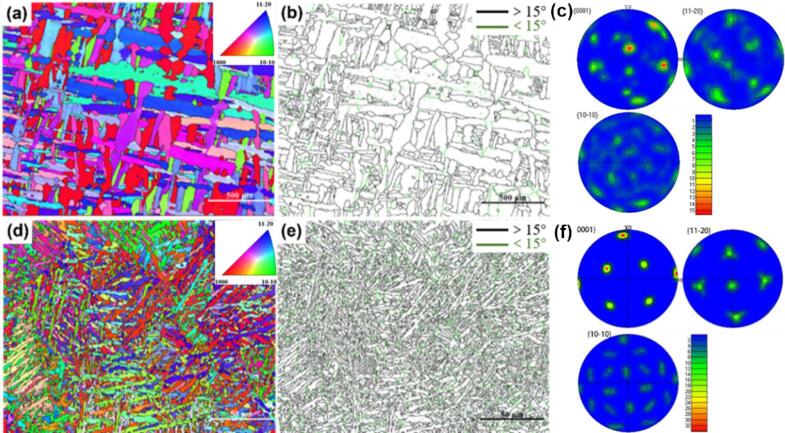
EBSD graphs of as-cast TC4 (a-c) and LPBF TC4 (d-f) alloys: (a, d) inverse pole figure, (b, e) grain size histogram, (c, f) pole figures.

Fig. 3 illustrates the EBSD results for cast TC4 and LPBF TC4 alloys. In Fig. 3(a) and (d), the grain orientation is more homogeneous for both alloys, while in contrast the alloy seems to be slightly more pronounced in the <1000> orientation. Fig. 3(b) and (e) show that the as-cast alloys have more complex grain types with columnar, dendritic, and equiaxial crystals, and the difference in grain size is more pronounced, whereas the LPBF TC4 alloys are basically dendritic, with smaller and uniform grain size. Fig. 3(c) and (f) display the pole figures (PFs) of as-cast and LPBF TC4 alloys. Both the two samples exhibit {0001} basal texture, but the texture of the LPBF TC4 alloy is much stronger. With a maximum texture density of 34.23, the LPBF TC4 alloy exhibits a considerable enhancement compared to the as-cast TC4 alloy, which registers at 15.73. This phenomenon may arise from the extended duration of the alloy forming process, which results in accelerated grain growth along the <1000> direction parallel to the temperature gradient, thus fostering the development of a pronounced texture.

**Fig. 4:**
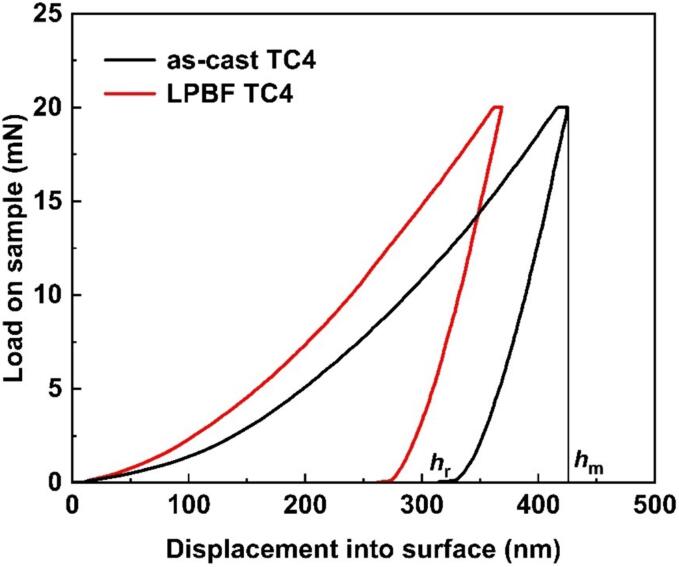
Load-displacement curves of as-cast TC4 and LPBF TC4 alloys.

Fig. 4 displays the nanoindentation load–displacement plots of the as-cast TC4 and LPBF TC4 alloys. In load–displacement curves, the elastic properties of materials can be scrutinized through the depth recovery ratio (*η*_h_), derived from the plots [36]. The calculation of *η*_h_ value can be made as follows:

(2) $$\eta_{\text{h}}=\frac{{\text{h}}_{\text{max}}\text{-}{\text{h}}_{\text{r}}}{{\text{h}}_{\text{max}}}$$

In which, *h*_max_ represents the maximum depth of penetration (nm), while *h*_r_ denotes the residual depth after unloading (nm). The application of a load of 20 mN yields that the *h*_max_ values for the as-cast TC4 and LPBF TC4 are 424.7 nm and 368.6 nm, respectively. Apparently, the *h*_max_ for as-cast TC4 is larger than that of the LPBF TC4, suggesting the higher hardness or elastic modulus of LPBF TC4. The elevated *η*_h_ value of the LPBF TC4 implies that the LPBF alloy possesses superior elastic properties in comparison to as-cast TC4 alloy. Qiao *at al.* [37] reported that materials with higher elastic properties displayed higher CE resistance. Thus, it can be inferred that LPBF TC4 may possess lower CE rate compared to as-cast TC4 alloy (See Table 3).

**Table 3:** Indentation parameters derived from Fig. 4.

	*h*_max_ (nm)	*h*_r_ (nm)	*η*_h_
as-cast TC4	424.7	314.5	0.26
LPBF-TC4	368.6	262.2	0.29

### 3.2 Mass loss

**Fig. 5:**
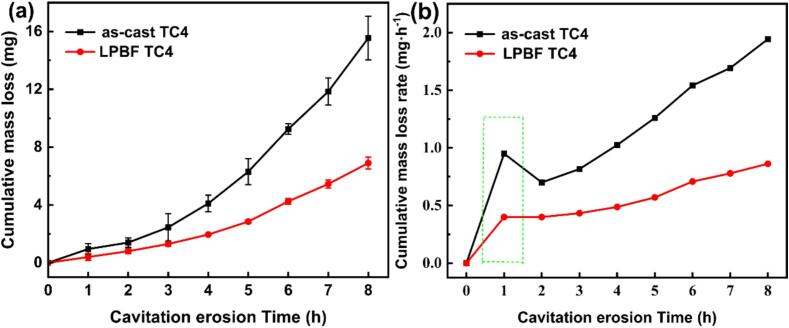
Cumulative mass loss (a) and cumulative mass loss rate (b) of as-cast TC4 and LPBF TC4 alloys exposed to 0.6 mol/L NaCl solution.

Fig. 5 demonstrates the changes of the cumulative mass loss and cumulative mass loss rate of both as-cast and LPBF TC4 alloys immersed in 0.6 mol/L NaCl solution at varying durations of CE. As the CE time extends, the two alloys exhibit an escalation in cumulative mass loss. In general, as-cast TC4 has a higher cumulative mass loss rate compared to LPBF TC4. The extension of CE time to 8 h, as depicted in Fig. 5a, results in the cumulative mass loss of as-cast TC4 reaching 15.55 mg, whereas for LPBF TC4, it is notably lower, 6.90 mg. Clearly, as-cast TC4 has 2.25 times higher cumulative mass loss than LPBF TC4. Meanwhile, as illustrated in Fig. 5b, the former cumulative mass loss rate experiences a pronounced surge to 0.95 mg/h over the CE period of 1 h. This phenomenon can be attributed to the impact of continuous CE treatment for 1 h, wherein the interplay between large and small pores gives rise to the formation of pits, consequently instigating material detachment. Analogous observations have been corroborated in the research conducted by Li and colleagues [34]. Similarly, there also exhibits an notable increase in the mass loss rate observed for the LPBF alloy under the same exposure period, but the value is considerably lower than the as-cast one [25]. The observations pertaining to cumulative mass loss and cumulative mass loss rate support that the LPBF TC4 specimen demonstrates a superior CE resistance in comparison to as-cast TC4 alloy.

### 3.3 CE evolution morphologies

**Fig. 6:**
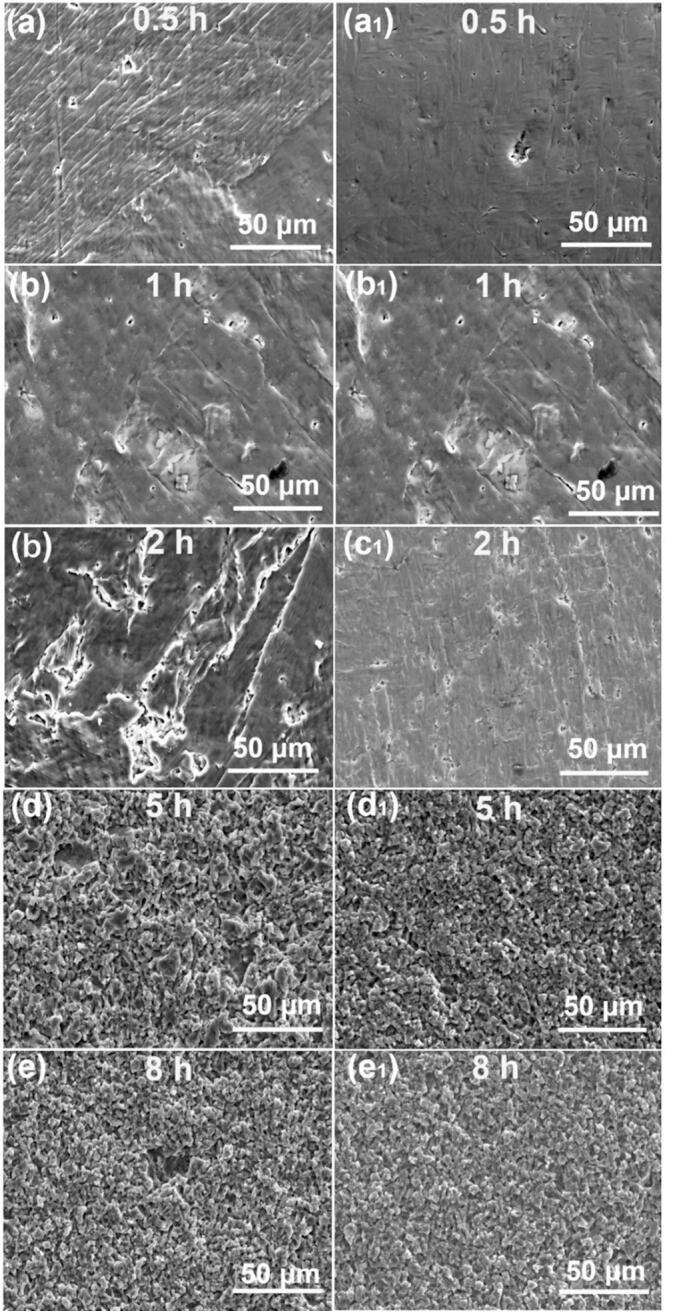
CE evolution morphologies of as-cast TC4 alloy at CE time of (a) 0.5 h, (b) 1 h, (c) 2 h, (d) 5 h and (e) 8 h; the CE evolution morphologies of LPBF TC4 alloy after CE for (a_1_) 0.5 h, (b_1_) 1 h, (c_1_) 2 h, (d_1_) 5 h and (e_1_) 8 h.

Fig. 6 depicts the surface morphologies of the as-cast TC4 and LPBF TC4 samples corresponding to different CE durations. After CE for 30 min, cavities, surface undulations and traces of slip lines are observed on the as-cast TC4 surface (Fig. 6a). Under these circumstances, the failure mode of as-cast TC4 alloy is similar to the stainless steel and CoCrFeNi high entropy alloy (HEA) [37], [38], [39]. The slip lines are distributed inside an individual grain in the same direction. In contrast, slip lines are absent in the adjacent grain, and this is mainly caused by the spatial orientation difference for the adjacent grains [40]. Because of the progressive extrusion induced by plastic deformation and the notch effects, material peeling occurs at the slip bands and grain boundaries (Fig. 6b). With extending CE time, severe plastic deformation occurs, and noticeable material loss is observed at grain boundaries and slip lines, as shown in Fig. 6c. There exhibits visible CE variation between the as-cast and the LPBF TC4 alloys due to the difference in crystal structure. At the same CE periods, a smaller number of slip lines are observed for LPBF TC4 (Fig. 6a1). The surface undulations and traces of slip lines on LPBF TC4 are not obvious, but small cavities exist on the surface (Fig. 6b1). Prolonging the CE time, the number of cavities increases and severe plastic deformation occurs (Fig. 6c1). Likewise, CE damage initiates either at grain boundaries or slip lines inside the grains. After a long CE period (5 h and 8 h), the mass loss rates of the as-cast TC4 and LPBF TC4 alloys increase with the CE time, and this is the typical characteristic of the acceleration stage in the CE process. A debris fracture mode is predominantly shown under the CE attack (Fig. 6d, d1, e and e_1_). The existence of micro-cracks and dimples on the eroded surface also demonstrates ductile fracture failure. Similar results were reported in the published work [41].

### 3.4 Electrochemical corrosion response

**Fig. 7:**
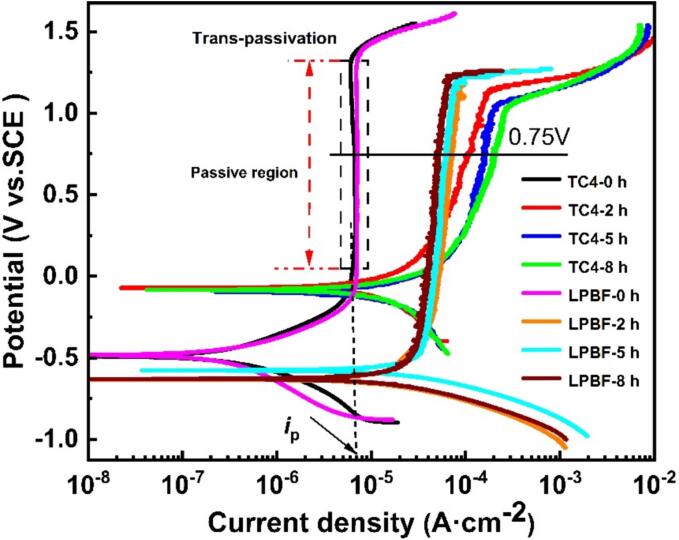
Potentiodynamic polarization (PDP) curves of as-cast and LPBF TC4 alloys after different CE times in 0.6 mol/L NaCl solution.

Fig. 7 presents the PDP curves of as-cast TC4 and LPBF TC4 at various CE durations in 0.6 mol/L NaCl solution. Under static condition (0 h), the corrosion responses of as-cast TC4 and LPBF TC4 are similar, indicating the similar electrochemical response. Table 4 lists the electrochemical parameters derived from Fig. 7. Both alloys exhibit typically spontaneously passive behavior and a broad passive domain. However, the Flade potential (*E*_f_) of as-cast TC4 surpasses that of LPBF TC4, indicating an easier tendency to establish passivation/repassivation for LPBF TC4 [22], [42]. It is noteworthy that the LPBF TC4 demonstrates a notably higher passive current density (*i*_p_) of 7.12 × 10^−6^ A·cm^−2^, about 10 times of the results reported by Dai *et al*. [43] in the same solution (8.41 × 10^−7^ A·cm^−2^). Meanwhile, some transient current peaks indicative of metastable pitting initiation and annihilation are observed in the passive region of LPBF TC4 [44]. The presence of metastable α′-martensite is responsible for the higher *i*_p_ and the presence of transient current peaks [43]. The transpassive behavior is often observed for pure titanium [45] and its alloys [8], [22], [43] in Cl^–^ containing solutions. In this work, the transpassivation potential (*E*_tr_) is observed from 1.2 V_SCE_ to 1.5 V_SCE_. It is evident that CE significantly impacts the electrochemical behavior of the two TC4 alloys. CE leads to the positive shift by ∼400 mV in *E*_corr_ for as-cast TC4, and the significant increase of *i*_corr_, which is more than dozens of times higher than that under static condition. The *E*_tr_ representing the stability of passive film, diminishes with prolonged CE time. After CE test, the passive region of as-cast TC4 regresses prominently. However, CE leads to the negative shift in *E*_corr_ for LPBF TC4 and the substantial increase of *i*_corr_. The *i*_corr_ and *i*_p_ for both two alloys increase with the increasing CE time. Therefore, it can be concluded that CE degrades the protection of the passive films and accelerates the corrosion dissolution of the two alloys.

**Table 4:** Electrochemical parameters of *E*_corr_ and *i*_p_ of the two TC4 alloys.

**Samples**	**CE time (h)**	***E*_corr_ (mV vs. SCE)**	***i*_p_ (A⋅cm^−2^)**
As-cast TC4	0	−489.5	6.67 × 10^−6^
	2	−71.52	1.08 × 10^−4^
	5	−88.91	1.63 × 10^−4^
	8	−83.28	2.04 × 10^−4^

LPBF TC4	0	−483.1	7.12 × 10^−6^
	2	−632.2	7.06 × 10^−5^
	5	−575.5	6.19 × 10^−5^
	8	−628.7	5.02 × 10^−5^

Apparently, the *E*_f_ of as-cast TC4 is higher than that LPBF TC4, as shown in Fig. 7, suggesting an easier tendency to spontaneous passivation for LPBF TC4 [22], [42]. The passivation ability of metallic materials can be evaluated using Eqs. (3), (4) [46]:

(3) $$i=\text{A}\times t^{-k}$$

**Fig. 8:**
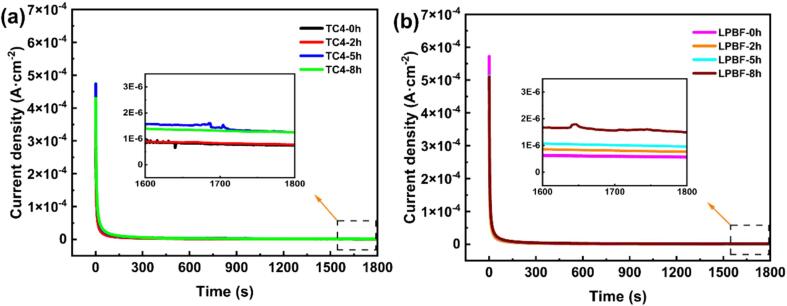
Plots of *i*–*t* response of the two TC4 alloys polarized at 0.2 V_SCE_ in 0.6 mol/L NaCl solution: (a) as-cast TC4; (b) LPBF TC4.

**Fig. 9:**
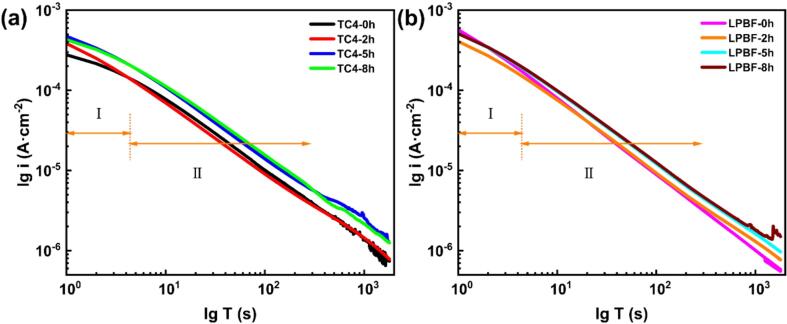
Log*i*–log*t* plots on the as-cast TC4 (a) and LPBF TC4 (b) in 0.6 mol/L NaCl solution.

**Fig. 10:**
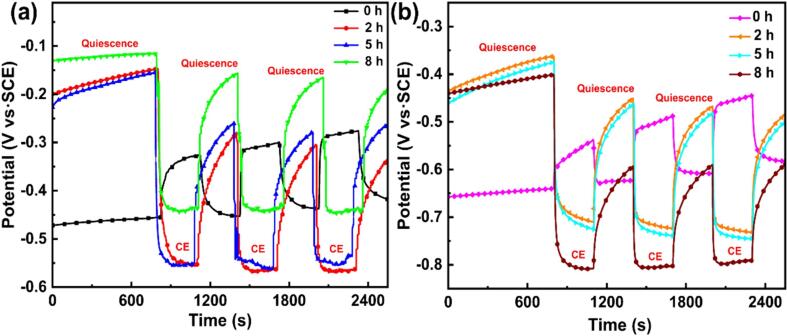
*E*_corr_ of the as-cast TC4 (a) and LPBF TC4 (b) alloys after alternating CE and quiescence cycles in 0.6 mol/L NaCl solution.

(4) lgi=lgA-klgt

where *i* is the current density, A is a constant, *t* is the time of the potentiostatic polarization test, and *k* is the passivation index. As for the results of potentiostatic polarization (See in Fig. 8), the decay of current density in early stage, i.e., in region I, is attributed to the alteration of the native oxide layer, which occurs initially during the potentiodynamic scanning to 1 V_SCE_ [47]. The decay rate of LPBF TC4 is relatively higher than that of as-cast TC4, indicating the higher passivity capability, as can be seen in Fig. 9. Additionally, the data presented in Table 5 reveals that the obtained *k* values for both alloys decline with increasing CE time, signifying a detrimental impact on the repairing capacity of the damaged passive film [48], [49]. After CE for the same period, the *k* value of LPBF TC4 is higher compared with that of as-cast TC4, suggesting LPBF TC4 has a stronger repassivation ability and a more stable passive film. The potentiodynamic polarization and potentiostatic polarization results show that LPBF TC4 has a better prospect of use and future development in CE environment. Fig. 10 exhibits the *E*_corr_ outcomes of both the as-cast and LPBF TC4 alloys after CE for different periods under alternative quiescence and CE conditions in 0.6 mol/L NaCl solution. For both samples without pre-CE, the *E*_corr_ gradually shifts towards a positive potential over the course of 800 s, and this result is consistent with that reported in the literature [50]. When CE starts, a positive shift of the *E*_corr_ is also observed with extending CE time. He *et al.* [51] attributed this phenomenon to the increased oxygen mass transport facilitated by CE, thereby accelerating the cathodic reaction process. The above result indicates that the passive films developed on both specimens are intact, and the impact force induced by CE is insufficient to cause the film rupture in a short period of CE time. Upon termination of CE, the *E*_corr_ experiences a rapid decline, followed by the establishment of stabilization at a consistent value. Notably, for the LPBF specimen, the *E*_corr_ under the quiescence condition rises gradually with increasing quiescence-CE duration, demonstrating the improved film protectiveness. However, for specimens subjected to CE durations of 2 h, 5 h and 8 h, an *E*_corr_ variation, which is different from that of the initial samples, appears under the CE condition. Once CE starts, the *E*_corr_ decreases sharply and then slightly, and it finally contains relatively constant until CE is terminated. The decrease in *E*_corr_ is associated with the rupture of passive film, triggered by the impact of CE [52], [53]. Moreover, the appearance of cracks and cavities hinders the development of a dense and highly protective passive film. Thus, the passive film can be easily damaged and the *E*_corr_ decreased correspondingly under the CE condition. Once the CE stops, the passive film can reestablish itself on the surface, leading to an increase in the *E*_corr_. In the case of pre-CE as-cast samples, it is noteworthy that the *E*_corr_ demonstrates an increase concomitant with extending the CE period, irrespective of whether subjected to CE or maintained in a quiescent state. Conversely, an opposing pattern is discerned in pre-CE LPBF samples. Following an extended period of CE, a conspicuous detachment of material is observed on the as-cast sample surface, wherein the flawed surface undergoes removal. Consequently, the *E*_corr_ experiences an elevation attributable to the heightened passivation capacity. This finding aligns with the observations made by Li *et al* [34]. Furthermore, the CE-induced compressive stress on the material surface is instrumental in enhancing the passivation ability [54], consequently leading to a proportional increase in the *E*_corr_ value.

**Table 5:** *k* value for two TC4 alloys derived from potentiostatic polarization plots.

	**0 h**	**2 h**	**5 h**	**8 h**
As-cast TC4	0.93	0.88	0.88	0.87
LPBF TC4	0.95	0.92	0.91	0.9

**Fig. 11:**
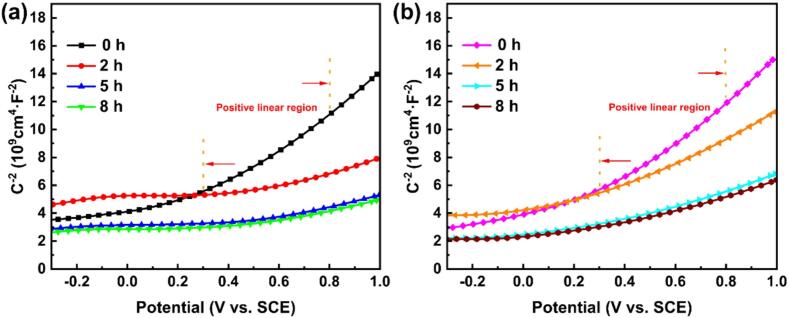
Mott-Schottky curves of the tested alloys after different CE times in 0.6 mol/L NaCl solution: (a) as-cast TC4, (b) LPBF TC4.

To elucidate the influence of CE duration on the semiconductor properties of the passive films formed on both alloys, Mott-Schottky (M-S) measurements are performed and their results are depicted in Fig. 11. Based on the M-S equation [55], the correlation between the space-charge layer (*C*) and the semiconductor film can be delineated as follows:

(5) $$\frac{\text{1}}{C^{\text{2}}}=\frac{\text{2}}{\varepsilon_{\text{r}}\varepsilon_{\text{0}}\text{e}N_{\text{D}}}\left(E-E_{\mathrm{FB}}-\frac{\mathrm{kT}}{\text{e}}\right)$$

In which, *ε*_r_ is the dielectric constant of the passive film, *ε*_0_ denotes vacuum permittivity (8.85 × 10^−14^ F/cm), *e* is electron charge (1.60 × 10^−19^ C), *N*_D_ represents donor density, *k* refers to Boltzmann constant (1.38 × 10^−23^ J·mol^−1^·K^−1^), *T* is temperature (K), *E* and *E*_FB_ is applied potential and flat band potential, respectively. Generally, a value of *ε*_r_ = 60 has been adopted based on the findings of Xu *et al.* [30] and Qiao *et al.* [56]. This choice is justified by the predominant composition of TiO_2_ in the passive films formed on pure titanium and its alloys.

**Fig. 12:**
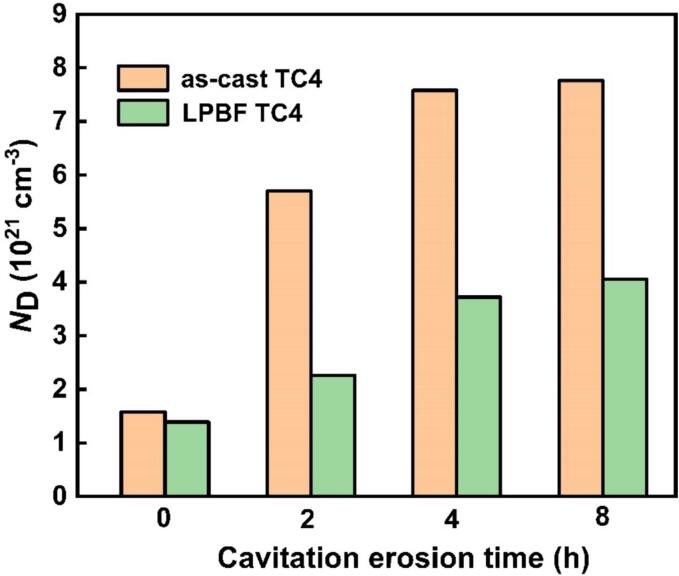
*N*_D_ values of the passive films formed on as-cast TC4 and LPBF TC4 alloys in 0.6 mol/L NaCl solution with different CE times.

As displayed in Fig. 11, both as-cast TC4 and LPBF TC4 present n-type semiconductor behavior, regardless of the CE time. These results are consistent with the M-S characteristic of TC4 fabricated by EBM [57], commercial TC4 [56] and commercial TA2 [45]. The prevalent electronic charge carriers within the passive films are oxygen vacancies (${\text{V}}_{\text{O}}^{\cdot \cdot }$) rather than cation interstitials (Ti^3+^), owing to their lower formation energy [30], [55]. The number of ${\text{V}}_{\text{O}}^{\cdot \cdot }$, i.e., *N*_D_, in the passive film intricately linked to the corrosion resistance of the metallic materials [44], [45]. Therefore, the *N*_D_ values of as-cast TC4 and LPBF TC4 in 0.6 mol/L NaCl solution with varying CE durations can be obtained from the slope of the linear region in the M-S curve, as depicted in Fig. 12. The implosion of bubbles induced by CE can alter the characteristics of the passive film [58].

Evidently, the quantity of oxygen vacancies (${\text{V}}_{\text{O}}^{\cdot \cdot }$) in the passive films of both alloys escalates with prolonged CE time. This phenomenon manifests as a result of the facilitation of flawed and disorganized passive film formation during prolonged cathodic electrolysis durations [30], [59], [60]. Comparable values for the donor density have been reported previously [61], and the trend of increasing *N*_D_ with CE time has been reported by Xu *et al.* [30]. At the same CE time, the calculated *N*_D_ value for as-cast TC4 surpasses that of LPBF TC4. As the CE time progresses (i.e., from 0 h to 8 h), the values of *N*_D_ for as-cast TC4 increase from 1.58 × 10^21^ cm^−3^ to 7.76 × 10^21^ cm^−3^, whereas only a marginal increase in the values of *N*_D_ for LPBF TC4 from 1.39 × 10^21^ cm^−3^ to 4.06 × 10^21^ cm^−3^. Typically, a lower *N*_D_ value results in reduced electron transport and conductivity, leading to slower electrochemical reactions [60].

### 3.5 Composition of passive films

**Fig. 13:**
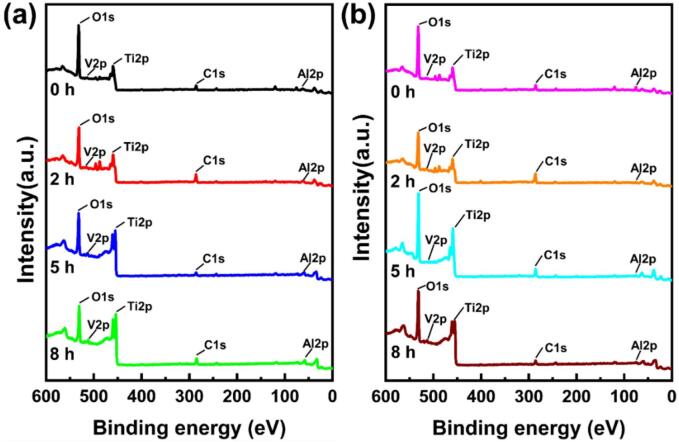
XPS full spectra of the surface passive film of as-cast TC4 (a) and LPBF TC4 (b).

**Fig. 14:**
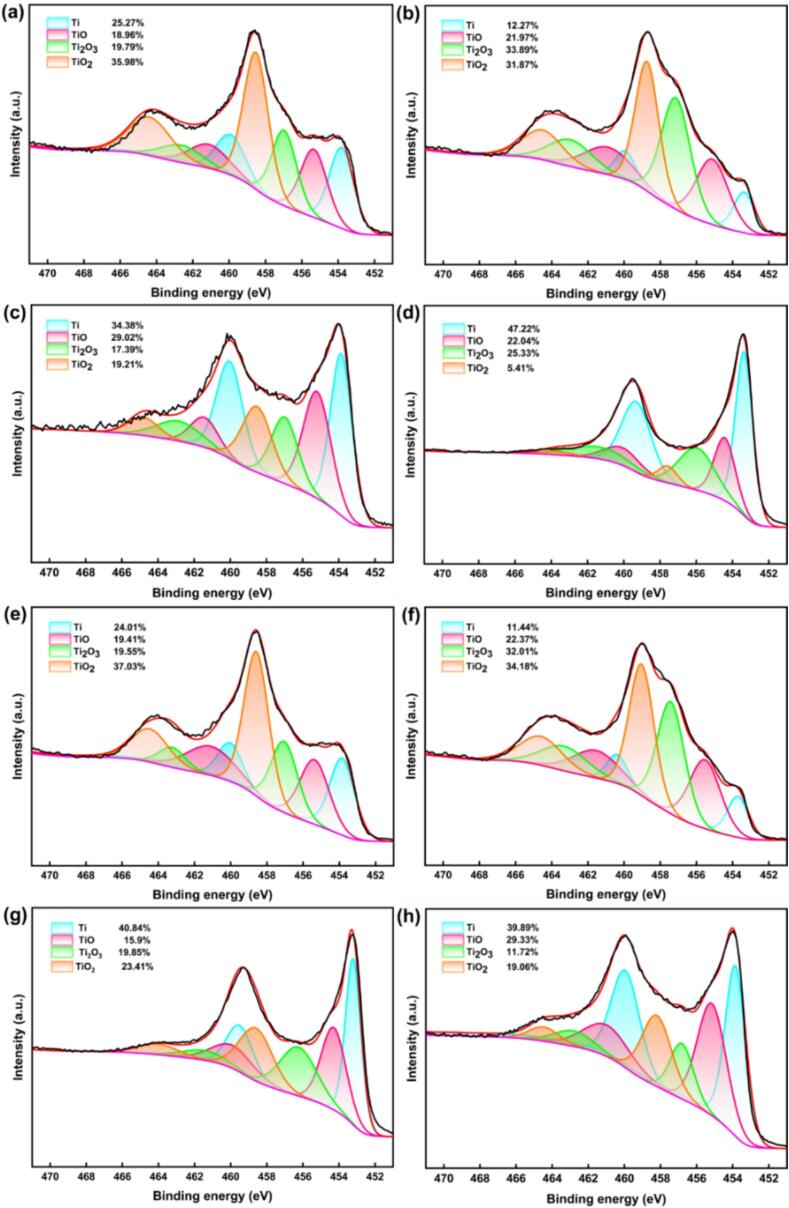
XPS spectra of Ti 3/2p in passive films of as-cast TC4 (a, b, c, d) and LPBF TC4 alloys (e, f, g, h) with CE time of 0 h, 2 h, 5 h, and 8 h.

**Fig. 15:**
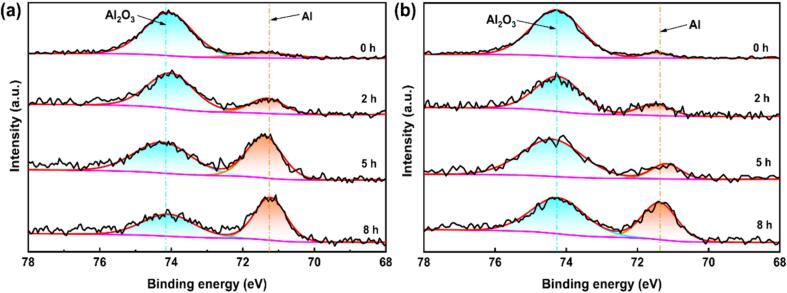
XPS spectra of Al2p in passive films of as-cast TC4 (a) and LPBF TC4 alloys (b) with CE time of 0 h, 2 h, 5 h, and 8 h.

The protective ability of the passive film is greatly influenced by the composition of oxide films. The full XPS spectra of the surface passive layers of these two TC4 alloys are shown in Fig. 13. After different CE times, Ti, Al, O, V are detected on the surfaces of the specimens. The O content of the passive film decreases with the increasing CE time. For the specimens without CE attack, TiO_2_ is the predominant component of the passive film, and its content reaches 35.98 % and 37.03 % for the as-cast and LPBF TC4 alloys, respectively. After 2 h CE, the content of TiO_2_ within the passive film diminishes, displaying 31.87 % and 34.18 % for the as-cast and the LPBF samples, respectively. After 5 h CE, the content of TiO_2_ inside the passive film is 19.21 % for the as-cast specimen and 23.41 % for the LPBF specimen. Upon completion of the CE period, the passive film on the cast surface exhibits a diminished TiO_2_ content and a notably elevated Ti (0) content. For the LPBF sample, the passive film contains a TiO_2_ content of 19.06 % and a Ti (0) content of 39.89 %. The content of Ti (0) increases with increasing sputtering depth. For specimens subjected to 5 h CE, the Ti (0) content on the surface reaches 34.38 % for the as-cast and 40.84 % for the LPBF sample, with a sputtering time of 20 s. These findings suggest that the passive films are very thin for both the two TC4 alloys. TiO_2_ is the most stable oxide of Ti. Generally, the effectiveness of the passive film enhances with the increasing TiO_2_ content. As shown in Fig. 14, the Ti O_2_ content decreases with the CE time, implying the decreased corrosion resistance. At the end of the same CE period, the passive film on the LPBF sample exhibited a higher TiO_2_ content, showcasing superior corrosion resistance compared to the as-cast sample.For the specimens without CE attack, Al_2_O_3_ is identified within the passive film, characterized by a minimal Al content [62], [63]. With the increasing CE time, the Al_2_O_3_ content also decreases, and the Al (0) content rises (Fig. 15). After CE for 5 h, the Al_2_O_3_ content reaches a very low value. This finding aligns with the result of Ti element presented above, underscoring the gradual decline in the protective properties of the passive films (See Table 6).

**Table 6:** Specific composition of passive film formed on as-cast TC4 alloy and LPBF TC4 alloy after different CE times.

**Sample**	**CE time (h)**	**Al**	**V**	**O**	**Ti**
as-cast TC4	0	15.68 %	0.24 %	63.57 %	20.51 %
	2	3.85 %	2.16 %	65.05 %	28.95 %
	5	10.08 %	13.32 %	44.55 %	32.05 %

LPBF TC4	8	4.84 %	12.59 %	43.28 %	39.30 %
	0	15.55 %	0.1 %	63.75 %	20.59 %
	2	5.01 %	1.73 %	64.50 %	28.76 %
	5	3.37 %	0.07 %	65.27 %	31.29 %
	8	4.29 %	14.37 %	47.82 %	33.52 %

### 3.6 CE damage mechanism

**Fig. 16:**
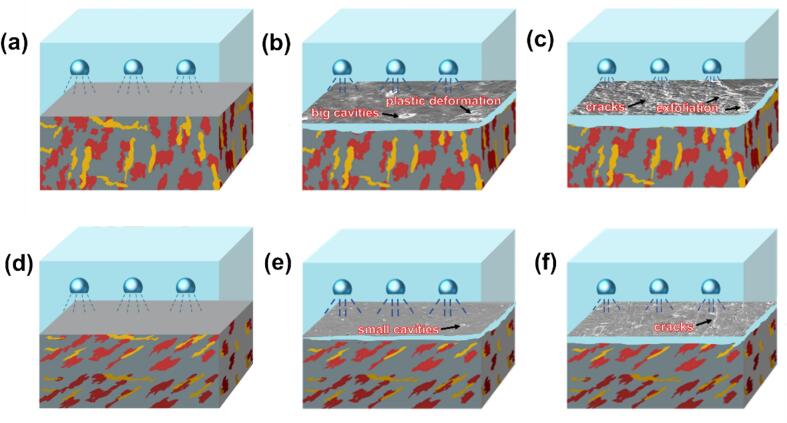
Schematic diagram of CE damage in cast TC4 (a, b, c) and LPBF TC4 (d, e, f), crack formation (a, d), crack extension (b, e), specimen spalling (c, f).

The schematic diagrams illustrating the CE damage process for both as-cast TC4 and LPBF TC4 alloys are presented in Fig. 16. During the initial CE period, depicted in Fig. 16(b) and (e), plastic deformation of the material surface triggers the formation of cavities. The proliferation of these cavities subsequently initiates and propagates cracks. Notably, grain refinement proves instrumental in retarding crack formation and propagation, thereby endowing the LPBF sample with superior CE resistance compared to its cast counterpart. Therefore, the quantity of cavities and cracks present on the surface of LPBF specimens is notably lower compared to that of cast specimens. Subsequently, in Fig. 16(c) and (f), the repetitive occurrence of bubble formation and collapse exerts stress on the material surface, causing cracks to propagate along the boundaries. As cracks continue to expand and interconnect, the alloy experiences spalling, leading to the creation of larger voids and the disappearance of numerous cracks. As anticipated, the refined and homogeneous microstructure effectively inhibits both the initiation and advancement of cracks, underscoring the capability of LPBF technology to bolster the CE resistance of titanium alloys.

## 4 Conclusions

In this study, a comparative investigation was conducted on the CE-corrosion behavior of LPBF TC4 and as-cast TC4 in 0.6 mol/L NaCl solution. The microstructure of as-cast TC4 manifested as a pine needle shape and comprised coarse-grained β-phase and a limited volume fraction of α-phase, while LPBF TC4 exhibited a rectangular checkerboard-like pattern with smaller grain size. In terms of CE resistance, LPBF TC4 outperformed the as-cast counterpart, exhibiting an approximately 2.25 times lower cumulative mass loss after 8 h CE. This enhanced CE resistance in LPBF TC4 could be ascribed to the refined grain structure and reduced metallurgical defects, thereby bolstering CE resistance and impeding surface damage processes. Results from the OCP testing in alternating CE and quiescent states suggested that, in the initial stages of CE, both LPBF TC4 and as-cast TC4 experienced a rapid potential decrease. Subsequently, with increasing CE time, the potential demonstrates an escalating trend, signifying a gradual reduction in repassivation ability. The initial rise in OCP during the early CE stages was primarily attributed to accelerated oxygen transfer. As CE progressed, the substantial decrease in OCP for both LPBF TC4 and as-cast TC4 was closely linked to the deterioration of the passive film. Overall, LPBF technology can improve the CE resistance of TC4 and extend its service life, and thereby facilitating the sustainable utilization and development of resources.

## CRediT authorship contribution statement

**Zhou Yang:** Investigation, Formal analysis. **Liang Li:** Investigation, Formal analysis. **Yanxin Qiao:** Writing – review & editing, Supervision, Conceptualization. **Chengtao Li:** Investigation, Formal analysis. **Lianmin Zhang:** Writing – review & editing, Supervision, Conceptualization. **Jie Cui:** Investigation, Formal analysis. **Dechun Ren:** Writing – review & editing, Supervision, Conceptualization. **Haibin Ji:** Writing – review & editing, Supervision. **Yugui Zheng:** Writing – review & editing, Supervision.

## Declaration of competing interest

The authors declare that they have no known competing financial interests or personal relationships that could have appeared to influence the work reported in this paper.

## Data Availability

Data will be made available on request.
